# Super-Resolution Scanning Transmission X-Ray Imaging Using Single Biconcave Parabolic Refractive Lens Array

**DOI:** 10.1038/s41598-019-50869-8

**Published:** 2019-10-07

**Authors:** T. Mamyrbayev, K. Ikematsu, P. Meyer, A. Ershov, A. Momose, J. Mohr

**Affiliations:** 1 Institute of Microstructure Technology, Karlsruhe Institute of Technology, Karlsruhe, Germany; 2 Institute of Multidisciplinary Research for Advanced Materials, Tohoku University, Sendai, Japan; 3 Institute for Photon Science and Synchrotron Radiation, Karlsruhe Institute of Technology, Karlsruhe, Germany

**Keywords:** Optics and photonics, Engineering

## Abstract

A new super resolution imaging technique which potentially enables sub-µm spatial resolution, using a detector of pixels much larger than the spatial resolution, is proposed. The method utilizes sample scanning through a large number of identical X-ray microprobes periodically spaced (the period corresponds to a multiple of the pixel size), which reduces drastically the scanning time. The information about the sample illuminated by the microprobes is stored by large detector pixels. Using these data and sample position information, a super-resolution image reconstruction is performed. With a one-dimensional (1D) high aspect ratio nickel single lens array designed for theoretically expected sub-µm microprobes at 17 keV and fabricated by deep X-ray lithography and electroforming technique, 2 µm X-ray microprobes with a period of 10 µm were achieved. We performed a first experiment at KARA synchrotron facility, and it was demonstrated that the smallest structure of a test pattern with a size of 1.5 µm could be easily resolved by using images generated from a detector having a pixel size of 10.4 µm. This new approach has a great potential for providing a new microscopic imaging modality with a large field of view and short scan time.

## Introduction

Many applications of X-ray imaging in material sciences need X-ray energies above 15 keV and spatial resolution at the micro- and nanoscale with a large field of view (FoV)^1–4^. The simplest approach to achieve a high spatial resolution is to use high resolution detectors, like X-ray CMOS and CCD, with densely packed tiny pixels coupled with magnifying optics. The effective pixel size can be as little as several hundred nanometers^5,6^. However, pixel size reduction by keeping the same pixel number decreases the FoV of an imaging system. Consequently, development of another method is necessary.

One of the powerful techniques for resolution enhancement is scanning transmission X-ray microscopy (SXTM), using a state-of-the-art nanoprobe available at high brilliance synchrotron facilities. It utilizes an optical element which creates a single focused X-ray beam, and an object is raster-scanned across the incoming beam^7,8^. The transmitted X-ray intensity is measured by a typically integrating detector at each scanning position. Nowadays, Fresnel zone plates (FZP), compound refractive lenses (CRL), reflection imaging mirrors or multilayer Laue lenses (MLLs) are used as focusing elements^9–12^. The highest spatial resolution achieved is below 10 nm and at the moment the FoV is up to 30 µm × 30 µm^13–15^.

Another approach to enhance spatial resolution is a super-resolution (SR) imaging technique. The main advantage of SR techniques is that the SR image reconstruction allows to generate high resolution (HR) images from experimentally measured multiple low resolution (LR) images without changing the hardware^16–25^.

Most of SR techniques can be categorized into two groups: frequency and spatial domain methods^26^. Mainly, frequency domain approaches are utilized if the global motion model is applied, i.e. when the detector (camera) is moving with the subpixel shift and the sample (scene) is fixed. The method is based on the registration of a series of aliased images based on their low frequency, aliasing free part. The HR image is reconstructed by cubic interpolation^27^. This technique was demonstrated in the soft X-ray region (1.8 keV) by utilizing an X-ray microscope based on FZP and an SR X-ray CCD camera using a piezo-driven sub-pixel shift mechanism integrated into the camera head^28^.

The benefit of the spatial domain SR approaches is the use of arbitrary motion models and especially non-global translation models, i.e. when the sample is moving and the detector is fixed. In X-ray imaging, the scanning of a sample with sub-pixel sized steps in front of the detector is preferable because it allows eliminating field inhomogeneity of the incident X-ray beam by flat field correction^29^. The first demonstration of this technique was realized by the raster-scanning of a sample in front of the single-photon counting LAMBDA detector without the use of optical elements. In this work, the spatial resolution of 10 µm was achieved^30^.

In this work, we propose a new super-resolution scanning transmission X-ray imaging (SRSTXI) technique which combines the advantages of STXM and SR imaging in the spatial domain.

The method utilizes sub-pixel sample scanning through a large number of identical X-ray microprobes periodically spaced, whereas the period corresponds to the pixel size of a low resolution pixel detector located behind the sample. The spatial resolution and FoV are determined by the microprobe size and the number of microprobes, respectively. The scan time is reduced in proportion to the number of microprobes. An array of microprobes is formed by an array of refractive optical elements. The space between microprobes needs to be equal to a multiple of the size of one pixel at the detector position, and each microprobe should impinge on the center of each pixel.

To demonstrate the proof of principle, we fabricated a one-dimensional nickel single lens array designed for 17 keV using deep X-ray lithography and electroforming. The number of lens elements was 250 and the period was 10 µm (detector pixel size 10.4 µm), allowing a FoV of 2.5 mm × 60 µm, where 60 µm corresponded to the current height of the lens array. The experiments were performed at the KARA IMAGE beamline. The sample was illuminated by the microprobes created by the nickel lens and scanned along the direction of the lens array. We carried out hard X-ray imaging using an in-house resolution test pattern (smallest feature is a 3 µm-thick gold line with a width of 1.5 µm).

## Imaging Principle and Design

**Figure 1:**
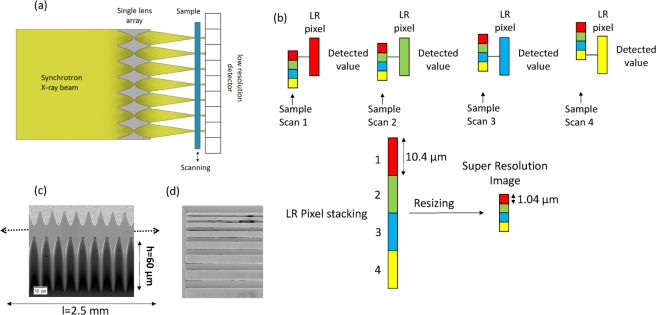
Representation of the super-resolution X-ray imaging method. (**a**) Experimental setup with a single lens array for X-ray absorption-contrast imaging. For the simplification of the scheme, only 7 single lens are shown. The 1D micro lens array is illuminated by synchrotron radiation through a double-crystal monochromator and generates an array of line focuses. The sample is placed in the focal plane and scanned as shown by the arrow with sub-pixel steps by a piezo stage. The detector is placed just behind the sample. (**b**) Image reconstruction method: at each sample scan position, the focal spots illuminate the sample and the information of local transmission is measured by LR pixels. Super-resolution image reconstruction is performed by stacking LR pixel values and resizing them to the original size. (**c**) SEM image of the fabricated single lens array via deep X-ray lithography and electroforming. The total length of the lens array is 2.5 mm, the height is 60 µm (**d**) SEM image of the resolution test pattern with the smallest feature of 1.5 µm gold lines with 3 µm height.

The super-resolution X-ray imaging principle is shown in Fig. 1a. A micro lens array is placed in the synchrotron beam and oriented vertically, expecting a focusing performance better than that when it is oriented horizontally. The lens array is illuminated by a monochromatic X-ray beam, and an array of line focuses is formed with a period equal to the imaging pixel period. The sample is placed in the focal plane of the lens array and scanned in the vertical direction. The sample is positioned near to the detector in order to avoid image blurring due to X-ray scattering and finite source size. Sample scanning is performed using a sub-pixel step defined by the size of the illumination spot and the Nyquist sampling theorem. At each sample scan position, the generated microprobes illuminate the sample and the information is stored in LR pixels (Fig. 1b). The number of LR images (N_LR_) for SR imaging technique is defined as a ratio of the space between microprobes (period of the lens array, P_LA_) and the stepping value (Step_Piezo_): ${N}_{LR}=\frac{{P}_{LA}}{Ste{p}_{Piezo}}$. The absorption image is defined by the ratio of the number of photons impinging on the LR pixel with and without the sample. Using these data, the super resolution image reconstruction is performed by stacking LR pixel values and resizing them to the original size. The SR image reconstruction is described in *Method section*.

The single lens array shown in Fig. 1c was designed, taking into account the constraints at KARA IMAGE beamline (KIT, Karlsruhe, Germany):

- the maximum X-ray energy is 17 keV (wavelength 0.73 Å), which restricts the lens material choice in order to minimize the absorption^31,32^;
- the source size (rms) is 0.93 mm × 0.0253 mm (horizontal × vertical),
- the beam size at the sample position (8 mm horizontal × 1 mm vertical) limits the length of the micro lens array,

The diffraction limited spot size (*d*_diff_) is defined by ${d}_{diff}=\frac{\lambda }{N.A.}\approx \frac{\lambda F}{{A}_{eff}}$, where 𝜆 is the wavelength, N.A. is the numerical aperture, *F* is a focal distance and *A*_*eff*_ is an effective aperture^33^. In order to achieve a smaller focal spot size, a smaller focal distance is preferable^34–36^. The focal distance is defined by *F* = *R*/2𝜎, where *R* is the radius of curvature of the lens and 𝛿 is the refractive index decrement of the lens material. Using X-ray lithography, only three materials could be chosen practically: nickel, gold and polymer. In this study, nickel was chosen as lens material due to its refractive index decrement of 6.16 × 10^−6^ at 17 keV, and less absorption comparing to gold^37^. Using polymer like SU-8, the transmission is much higher, but focusing by a single lens array is practically impossible because the refractive index decrement (9.23 × 10^−7^) is one order of magnitude smaller than the value for nickel.

The fabricated one dimensional nickel micro lens array presents the following parameters:

- length: 2.5 mm, which corresponds to 250 identical lens elements
- period: 10 µm,
- physical aperture: 8.95 ± 0.23 µm,
- radius of curvature: 0.83 ± 0.2 µm,
- an individual refractive X-ray lens consists of biconcave parabolic profiles^31–36,38^. The distance between parabola apexes is: 8.75 µm ± 0.17 µm
- transmission ratio at apexes: 0.686
- height: ca. 60 µm.

From experimental results, the distance and FWHM of the focal spot size were evaluated to be 67 mm and ca. 2 µm, respectively. Details on the fabrication process, characterization of fabricated structures using scanning electron microscope (SEM), roughness measurements using optical three-dimensional surface profiler, and X-ray characterization at KARA IMAGE beamline are described in *Method section*.

## Results

### Resolution test pattern

Since our X-ray imaging method utilizes a 1D single lens array, it is sensitive in only one dimension. Thus, a 1D resolution test pattern was fabricated at IMT KIT using electron beam lithography. A SEM image is shown in Fig. 1d. The test pattern consisted of a 2.4 µm thick titanium membrane with a gold structure of 3 ± 0.5 µm in thickness and a length of 1 mm on top of it. The titanium membrane is nearly transparent for 17 keV X-rays and the gold structure absorbs 51% of the X-rays. The membrane was fixed on an aluminum frame to keep it flat. The smallest feature in the test pattern had a width of 1.5 µm.

### Super-resolution X-ray imaging of resolution test pattern

The sample (test pattern) was placed in the focal plane (67 mm downstream of the lens array) and scanned across the X-ray focal lines. The sample was placed 1 cm in front of the detector which was the closest reasonable position in order not to hurt the detector. Sample scanning using a piezo stage (Physical Instruments, Germany) was performed with 1 µm steps within 10 µm. The scanning step is equal to half of the microprobe size (FWHM/2) due to the sampling theorem to perform oversampling. At each sample step, HR images were acquired by the detector. In the present experiment exposure time was 10 s to acquire one HR image due to low photon flux at the IMAGE beamline KARA synchrotron facility. The total scanning time is defined by the acquisition of the images with and without sample and corresponds to ca. 4 min (ten HR images with sample 100 s, ten HR images without sample 100 s, sample stepping ca. 50 s).

**Figure 2:**
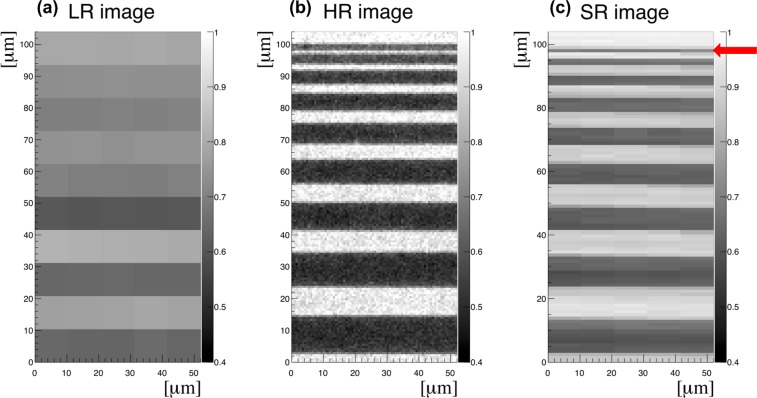
Super-resolution X-ray imaging of a resolution test pattern: (**a**) low resolution (LR) image (pixel size: 10.4 µm) formed from a high resolution HR image by binning, (**b**) high resolution (HR) image (pixel size: 0.65 µm) as measured. (**c**) Reconstructed super resolution (SR) image (pixel size: 1.04 µm) from ten LR images. The smallest feature of 1.5 µm is resolved (indicated by a red arrow). It is seen that the SR image is less noisy comparing to HR image, because SR image was made from ten HR images.

Each HR image consisted of 2560 × 2160 pixels (0.65 µm per pixel). A set of ten LR images with a size of 160 × 135 (10.4 µm per pixel) pixels were formed from HR images by 16 × 16 pixel binning. Super-resolution image reconstruction was performed by aligning LR pixel values and resizing the image to the original size. A result of SR imaging is presented in Fig. 2, where the smallest feature with a width of 1.5 µm of the test resolution pattern is resolved.

## Discussion

We demonstrated a new SR imaging technique by using a single lens array fabricated by deep X-ray lithography and electroforming. A gold line with a width of 1.5 µm could be resolved easily using a detector whose pixel size was 7 times larger than the structural width. As the spatial resolution of the presented method is restricted by the microprobe size, a sub-µm spatial resolution can be achieved via increasing the aperture and/or X-ray energy keeping the same focal distance which depends on the radius of curvature (current fabrication limit).

One of the main parameters for the successful SR image reconstruction in our technique is the stepping of the sample. This requires piezo stepping with high stability and no vibrations of the X-ray imaging system. Any imprecision during the scanning will lead to a reduction of the spatial resolution and artefacts in the super resolved image. The number of LR images for SR imaging technique is defined as a ratio of the space between microprobes and the stepping value according to the Nyquist Sampling Theorem.

The FoV of the presented method in the sample scanning direction is only limited by the length of the micro lens array, which could be increased up to 10 cm or even more easily in the future. However in the direction perpendicular to the sample scanning, it is limited by the height of the structures. Therefore, we are currently working to increase the height of the structures.

In this work, SRSTXI is utilised in one dimension (in the direction of sample scanning) due to the restriction of a one-dimensional lens array, in the second dimension the spatial resolution is defined by the low resolution detector pixel size. Therefore, reconstructed SR image is a 2D image with a pixel size of 1.04 µm in scanning direction and 10.4 µm in the horizontal direction. The method can be potentially applied for characterisation of micro- and nanowires, quality evaluation of X-ray gratings, investigation of the interfaces between composite materials.

Moreover, the proposed SR X-ray imaging technique can be expanded to a two-dimensional configuration. In this case, fabrication of 2D optical elements is needed to create a 2D point foci array and attain 2D super-resolution. One of the possibilities is to cross single lens arrays in orthogonal view^39–42^. This will help to create a 2D map of the sample.

## Summary

A super-resolution scanning transmission X-ray imaging (SRSTXI) technique was proposed and successfully demonstrated at the IMAGE beamline at KARA synchrotron facility with monochromatic X-rays of 17 keV using a newly designed and fabricated nickel micro lens array (period: 10 µm, radius of curvature: 0.83 µm, physical aperture: 9 µm, height: ca. 60 µm). Super-resolution image reconstruction was performed using ten low-resolution images and the 1.5 µm feature of a resolution test pattern could be easily resolved, which demonstrated a spatial resolution of 1.5 µm or even better. In comparison with conventional scanning transmission microscopy, in the present method, sample scanning was performed through 250 identical microprobes which drastically reduce the scanning time. Therefore, the SRSTXI is a promising technique for investigation of extended objects with the spatial resolution in sub-micron scale.

## Methods

### Single lens array fabrication

The single lens array was fabricated via deep X-ray lithography and electroforming. An X-ray sensitive polymer layer was spin coated onto the silicon substrate with a thickness of 525 µm covered with a 2.5 µm conductive seed layer (titanium with oxidized surface). The oxidation of the titanium surface increased the surface roughness and provided good adhesion of photoresist and electroplated metal structures. Polymethylmethacrylate (PMMA) was used as a positive resist with a thickness of 76 µm. A mask pattern was transferred onto the resist layer using highly collimated soft X-ray spectrum from a KARA synchrotron beamline (LITHO 1, energy range 2.2–3.3 keV). After exposure and development of the irradiated parts in a developer solution, a resist pattern was obtained. The nickel structures were created by an electroforming process; finally the resist was stripped^38–41,43–45^.

### Single lens array characterisation

#### Scanning electron microscopy

**Figure 3:**
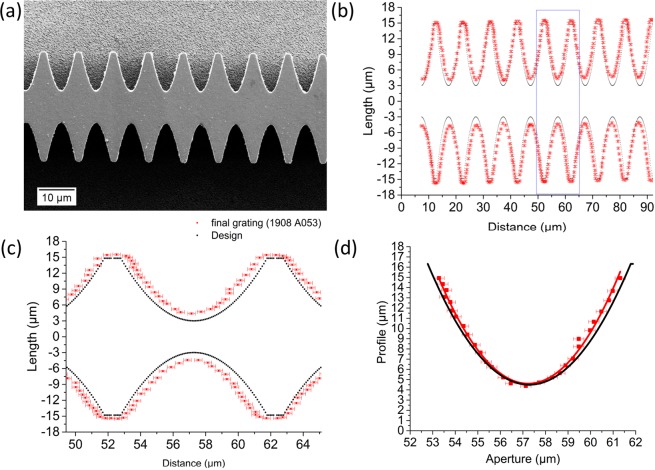
Analysis of the deviation of the fabricated structures from the design values: (**a**) SEM image of single refractive X-ray lens array fabricated by deep X-ray lithography and electroforming (top view). (**b**) Comparison of the fabricated structures (red) with the design values (black). The period of the structures is the same as the design value (10 µm). (**c**) Zoom-up of the blue rectangle in (**b**). The distance between parabola vertexes is enlarged from 6 µm (design value) to 8.5 µm (**d**) Designed parabolic profile (black curve) in comparison to the fabricated parabolic profile with parabolic curve fitting (red). The profile of fabricated structures is narrower than the designed one, and the radius of curvature changed from 0.86 µm (design value) to 0.83 µm.

A scanning electron microscope (SEM, Zeiss Supra 60 VP) operated at 5 kV was used to characterize the geometrical size of the single lens array (Fig. 3a). In Fig. 3b,c, a comparison of the designed structures (black dots) and the fabricated structures (red dots with error bars) is presented.

The form of the fabricated structures is in good agreement with the design. The small deviations are caused by polymer resist swelling during the electroforming process. The deviation of the fabricated structures from the designed form is shown in Fig. 3b. The distance between parabola apexes increased from 6 µm to 8.75 ± 0.17 µm and the physical aperture decreased from 9 µm to 8.95 ± 0.23 µm. The radius of curvature was analyzed to be 0.83 µm (design value was 0.86 µm). The deviations need to be minimized by further process optimization.

#### Surface roughness

The surface roughness of the fabricated single lens array with a small physical aperture and a radius of curvature in a sub-µm range is crucial. Although the surface roughness must be below less than 1 nm for high quality X-ray mirrors, the surface roughness required for refractive lens is below 100 nm^31^. We measured the roughness of the side wall of the fabricated nickel structures with an optical three-dimensional surface profiler (ContourGT, Brucker, USA) using the vertical scanning interferometry (VSI) mode with a green luminous source. The measured area was 130 µm × 170 µm, the average roughness (Ra) of the surface was evaluated to be 36 nm ± 5 nm using Vision-64 (Bruker) surface analysis software.

#### X-ray characterisation of micro lens array at KARA synchrotron facility

**Figure 4:**
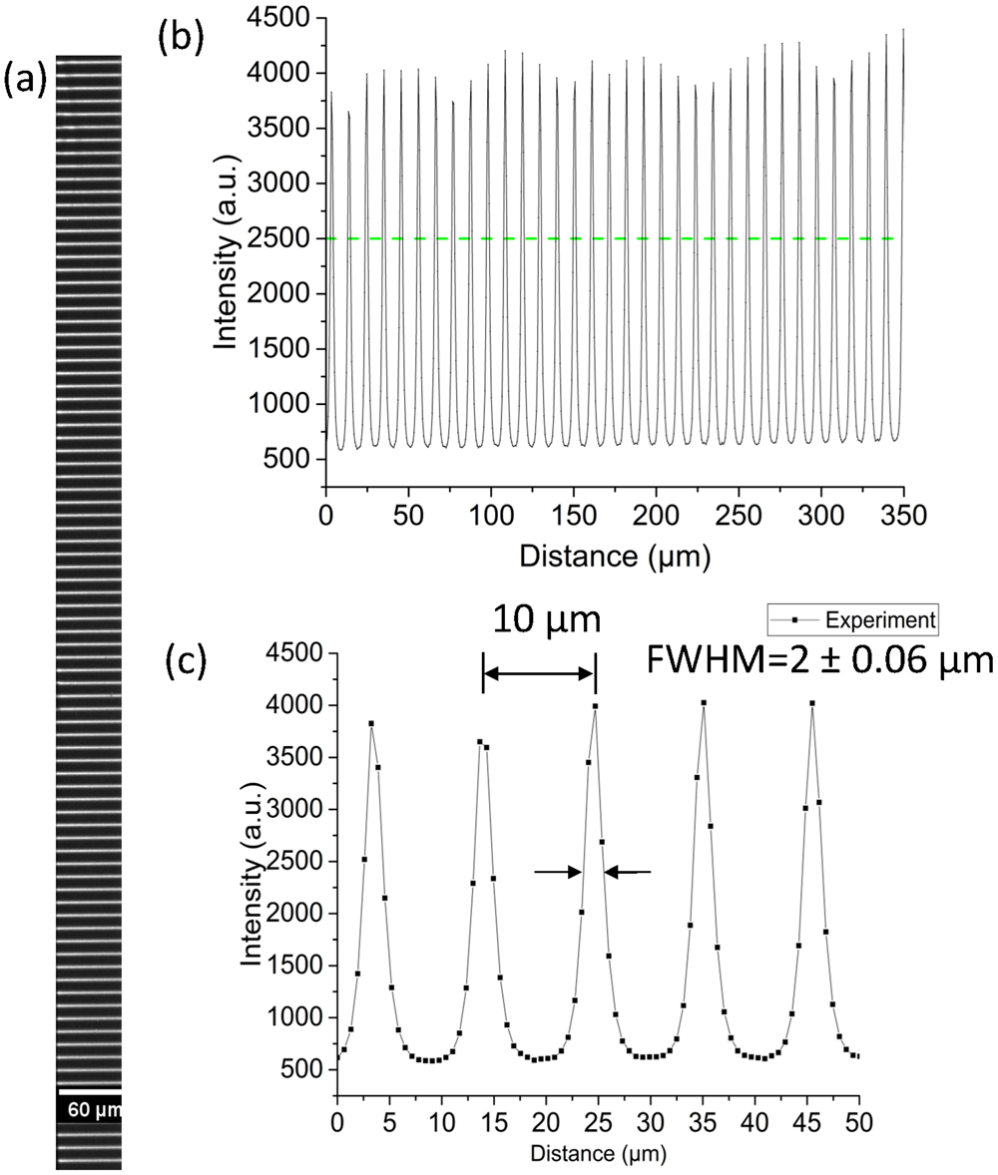
X-ray characterisation results: (**a**) X-ray focused lines generated by the micro lens array measured at 17 keV. The length of X-ray focused lines is 60 µm which is equal to the height of fabricated structures. (**b**) Profiles of line foci and (**c**) closed-up view. Green dashed line indicates the incoming beam intensity. All profiles are almost identical. Distance between foci is 10 µm and the size (FWHM) of the foci is 2 ± 0.06 µm.

An X-ray characterization of the micro lens array was performed at the wiggler IMAGE beamline at the KARA synchrotron radiation facility (KIT, Karlsruhe, Germany). A Si (111) double crystal monochromator, placed 15.5 m from the source was used to generate 17 keV monochromatic X-rays. To measure focal lines, a 74 µm LSO:Tb scintillator combined with 10 x magnification optics and a PCO.edge5.5 sCMOS camera (PCO AG, Kelheim, Germany) with an effective pixel size of 0.65 µm was used. The FoV of the detector were 1.66 mm (vertical) × 1.4 mm (horizontal). The micro lens array was oriented vertically since the source size in the vertical direction is much smaller than the horizontal one. The focal distance was determined by scanning the detector along the optical axis so that the intensity of focused beams was maximum, as shown in Fig. 4. The resulting focal distance was 67 mm, which was in a good agreement with the theoretical value (69 mm).

The measured focal line width was 2 µm ± 0.06 µm (FWHM), which was twice larger than the theoretical calculation. One of possible reasons for the spot size enlargement is the deviation of the fabricated parabolic profile from the desinged one (Fig. 3d). All focal lines had almost an identical form with a 10 µm period.

The gain was calculated as a ratio of the intensity of the focused beam and the incoming beam intensity within the size equal to the focused beam (60 µm × 2 µm). The measured gain was 1.4^31^.

### Super-resolution image reconstruction

Super-resolution image reconstruction^30^ was performed according to the following model: the intensity ${I}_{0}(x,y)$, generated by the one dimensional lens, is recorded by low resolution detector, where x and y are the pixel coordinates with a pixel size of *d*. Assuming that a sample is scanned through the focused beam in a vertical direction (y) with N steps which leads to an intensity change $I(x,y)$ in the detector plane due to absorption by the sample. The sample scanning should be performed within one low resolution detector pixel; therefore, the super resolution absorption contrast image ${A}_{SR}(x,\,Ny+n)$ is created as:

1 $${A}_{SR}(x,\,Ny+n)=\frac{I(x,y)}{{I}_{0}(x,y)}\cdot (S(x=d,\frac{n\cdot d}{N})),$$

n represents the step number, therefore, n = 1…N, and $S(d,\frac{n\cdot d}{N})$ is the scanning of the sample in the vertical direction y.

By scanning the sample with sub-pixel step through the intensity spot, low resolution pixel detects sub-pixel sized intensity changes which allows to perform SR reconstruction via sorting of the row vectors of the recorded LR images with a pixel size of d/N and stacking them into high resolution grid as it is described in Fig. 1b.

#### Data processing

All experimental raw data were flat-field and dark-field corrected. Low resolution images with a pixel size of 10.4 µm × 10.4 µm were generated by 16 × 16 pixel binning of high resolution images. Pixel binning and alignment were done using Numpy and SciKit image packages in Python programming language.

## Data Availability

The raw and processed data are available from the corresponding author on reasonable request.
